# A priori model independent inverse potential mapping: the impact of electrode positioning

**DOI:** 10.1007/s00392-015-0891-7

**Published:** 2015-07-28

**Authors:** A. W. Maurits van der Graaf, Pranav Bhagirath, Jacques de Hooge, Natasja M. S. de Groot, Marco J. W. Götte

**Affiliations:** Department of Cardiology, Haga Teaching Hospital, Leyweg 275, 2545 CH The Hague, The Netherlands; Department of Cardiology, Erasmus Medical Center, Rotterdam, The Netherlands

**Keywords:** Non-invasive imaging, Body surface potential mapping, Inverse procedures, Computational cardiac electrophysiology

## Abstract

**Introduction:**

In inverse potential mapping, local epicardial potentials are computed from recorded body surface potentials (BSP). When BSP are recorded with only a limited number of electrodes, in general biophysical a priori models are applied to facilitate the inverse computation. This study investigated the possibility of deriving epicardial potential information using only 62 torso electrodes in the absence of an a priori model.

**Methods:**

Computer simulations were used to determine the optimal in vivo positioning of 62 torso electrodes. Subsequently, three different electrode configurations, i.e., surrounding the thorax, concentrated precordial (30 mm inter-electrode distance) and super-concentrated precordial (20 mm inter-electrode distance) were used to record BSP from three healthy volunteers. Magnetic resonance imaging (MRI) was performed to register the electrode positions with respect to the anatomy of the patient. Epicardial potentials were inversely computed from the recorded BSP. In order to determine the reconstruction quality, the super-concentrated electrode configuration was applied in four patients with an implanted MRI-conditional pacemaker system. The distance between the position of the ventricular lead tip on MRI and the inversely reconstructed pacing site was determined.

**Results:**

The epicardial potential distribution reconstructed using the super-concentrated electrode configuration demonstrated the highest correlation (*R* = 0.98; *p* < 0.01) with the original epicardial source model. A mean localization error of 5.3 mm was found in the pacemaker patients.

**Conclusion:**

This study demonstrated the feasibility of deriving detailed anterior epicardial potential information using only 62 torso electrodes without the use of an a priori model.

**Electronic supplementary material:**

The online version of this article (doi:10.1007/s00392-015-0891-7) contains supplementary material, which is available to authorized users.

## Introduction

Inverse potential mapping (IPM) is a promising technique that may complement conventional invasive electrophysiological (EP) studies [1, 2]. In IPM, local epicardial potentials are inversely computed from recorded body surface potentials (BSP) [3]. Typically, 252 electrodes surrounding the thorax are used to record BSP [4, 5].

When a smaller number of recording electrodes is used, optimal electrode positioning is important. In the past, several studies have addressed this topic. Early studies focused on the detection and elimination of redundant information in the recorded BSP [6–10]. Later, biophysical a priori models, i.e., computer models that enable the in silico mimicking of in vivo conditions by using pre-programmed settings relating to physical properties, e.g., conduction velocity, fiber orientation, anisotropy, activation pathways, were introduced to compensate for the limited BSP data actually recorded [11]. In general, inverse procedures involving 64 or fewer electrodes always apply an a priori activation model.

The purpose of this study was to investigate the feasibility of IPM using only 62 torso electrodes in the absence of an a priori model. A simulation using 252 electrodes served as a reference for desired image quality. Simulations were performed using various electrode configurations. Three different electrode positions using 62 electrodes were subsequently applied on healthy volunteers to record BSP. From the recorded BSP, epicardial potentials were reconstructed. The amount of detail and the correlation with the original source model were assessed. To evaluate the localization error and size of the smallest visible detail, this mapping technique was applied in four patients with an implanted MRI-conditional DDD pacemaker system.

## Methods

### Computer simulations

#### 3D model

**Fig. 1:**
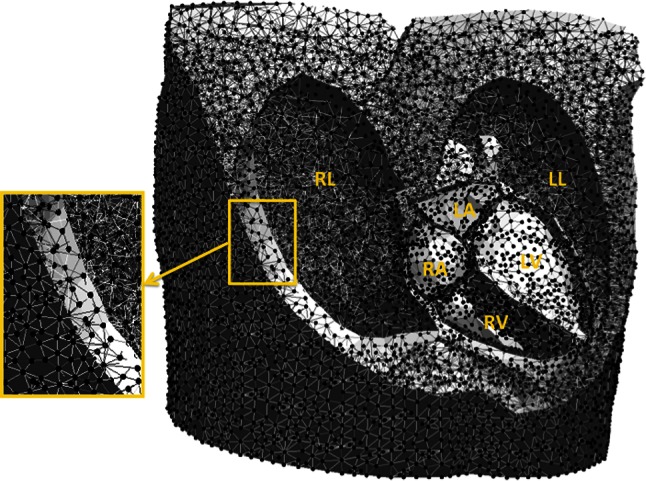
Example of a 3D volume mesh used for the simulations. This volume mesh was generated after segmentation of the thoracic organs on the MRI images. *RL* right lung, *LL* left lung, *RA* right atrium, *LA* left atrium, *RV* right ventricle and *LV* left ventricle

Simulations were performed using a 3D thorax model. This model was constructed after manual segmentation of different structures and organs on anatomic magnetic resonance imaging (MRI) images using custom written software. The model incorporated the whole-heart (including atria, ventricles, septum), liver, lungs, spleen, and torso surface. To each of these tissue elements conductivities were assigned as reported in literature (thorax: 0.2 S/m, lungs: 0.04 S/m, liver: 0.03 S/m and spleen: 0.04 S/m) [12]. Gmsh software [13] was used for the generation of a volume mesh, required for the simulations (Fig. 1).

#### Forward simulation

**Fig. 2:**
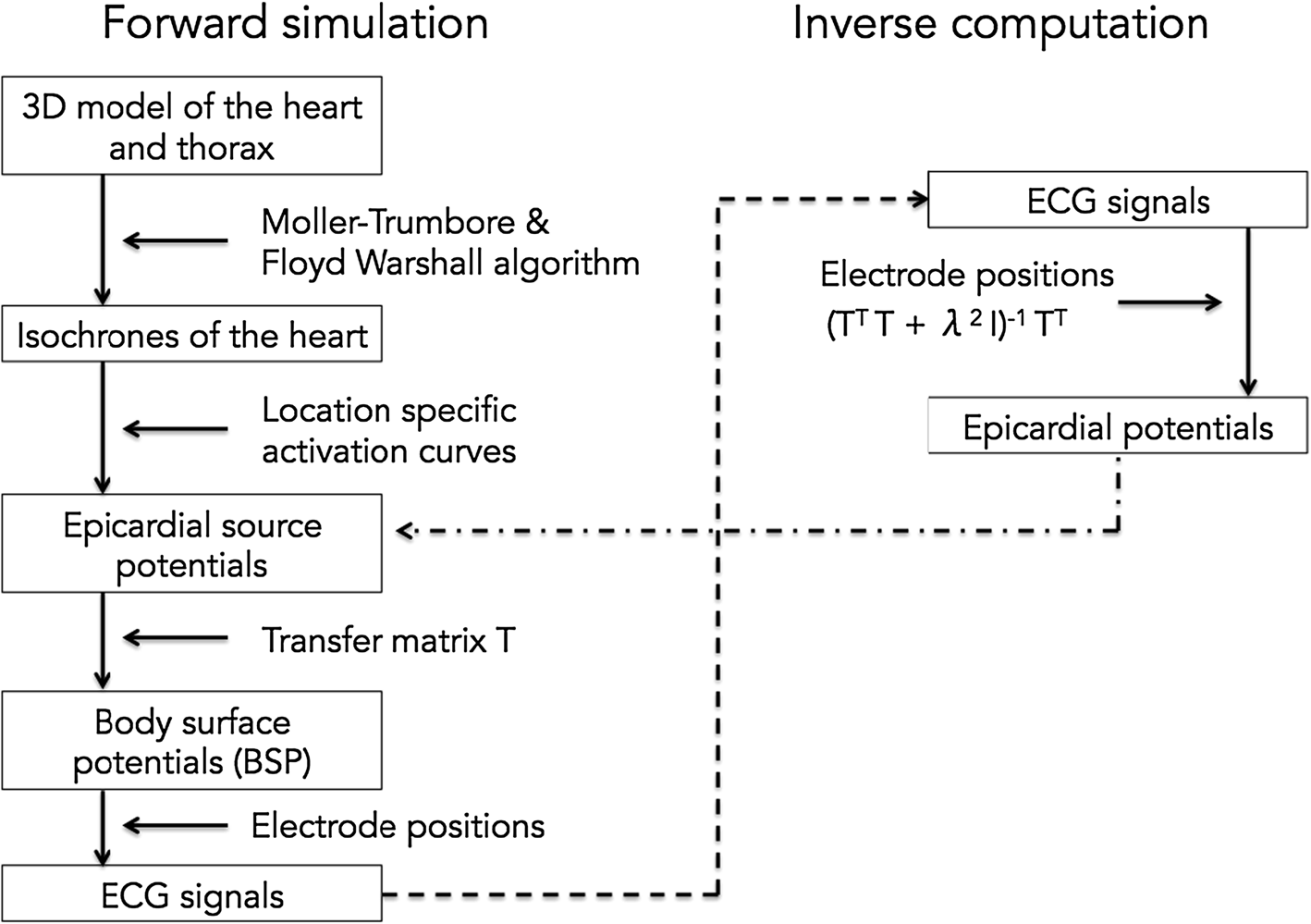
Flowchart visualizing the processes associated with forward simulations and inverse computations

Figure 2 provides a complete overview of the forward and inverse procedures used in this research, as described previously [14].

Propagation of electrical activity through the tissue elements of the whole-heart model was simulated using Moller-Trumbore [15] and Floyd-Warshall algorithms [16], yielding isochrones. From these isochrones, time dependent epicardial source potentials were computed by applying location specific activation curves. From the source potentials and in conjunction with the different tissue conductivities, BSP were computed by multiplication with the transfer matrix *T*. From these BSP, ECG potentials were computed by spatial sampling. In order to approximate real-life conditions, noise was added to the simulated ECG potentials with a signal-to-noise ratio (SNR) of 21 dB.

#### Inverse reconstruction of epicardial potentials from simulated ECG potentials

Epicardial potentials (*P*_epi_) were calculated from the ECG potentials (*P*_bs_) using *P*_epi_ = (*T*^*T*^*T* + *λ*^2^*I*)^−1^*T*^*T*^*P*_bs_ where *T* is the forward transfer matrix and *λ* is the regularisation strength, initially determined by simulation with patient-specific geometries. For all electrode configurations, the correlation between the initial source epicardial potentials (used for the simulation of ECG potentials) and the reconstructed epicardial potentials was computed. In addition, for all electrode configurations, correlation was calculated for 16 identical points on the anterior epicardium. These values were plotted in a graph to visualize correlation trends. To exclude systematic errors due to grid artefacts, all computations were performed on different grids.

**Fig. 3:**
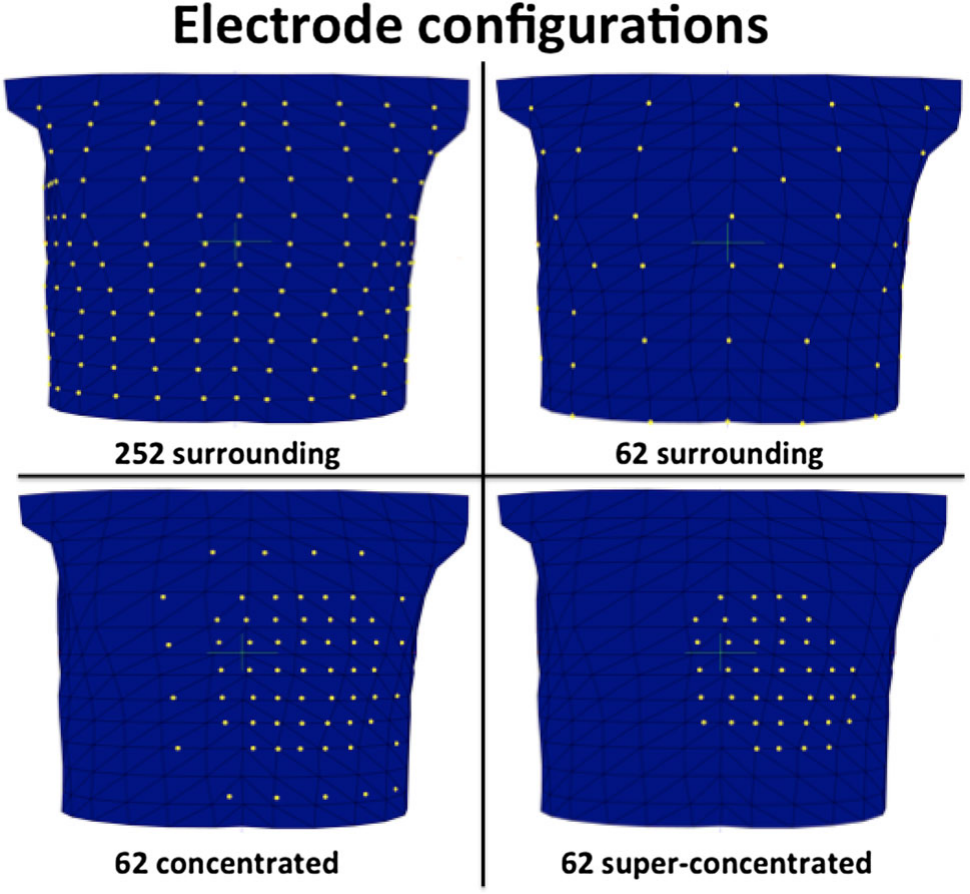
Anterior view of the thorax. Four different electrode configurations were used for the simulation part of the study: 252 electrodes surrounding the thorax, 62 surrounding the thorax, 62 concentrated (inter-electrode distance 30 mm) and super-concentrated (inter-electrode distance 20 mm). The *yellow* *markers* on the 3D thorax model represent the electrodes

Four different electrode configurations were used in the simulations (Fig. 3).

*Reference configuration* 252 electrodes surrounding the thorax. This configuration served as a reference, since this number of electrodes is the current standard in body surface potential mapping (BSPM).

*Configuration I* 62 electrodes surrounding the thorax.

*Configuration II* 62 concentrated (30 mm inter-electrode distance) electrodes directly overlaying the heart.

*Configuration III* 62 super-concentrated (20 mm inter-electrode distance) electrodes directly overlaying the heart.

### Inverse reconstruction of recorded human data

#### Study population

Three different electrode layouts were subsequently used to record data in three healthy volunteers (mean age 28 ± 1 years).

To evaluate the localization error and size of the smallest visible detail, four male patients (mean age 58 ± 12 years old) with an implanted MRI-conditional DDD pacemaker system (Advisa MRI™ Surescan^®^, Medtronic Inc., Minneapolis, MN, USA) and a structurally normal heart were enrolled. Patient characteristics are provided in Table 1. The RV lead tip was positioned either in the RV apex (2 patients), or in the right ventricular outflow tract (RVOT) (2 patients).

**Table 1:** Patient characteristics

Patient	Age (years)	Sex	RV lead tip location	Pacing indication	Relevant comorbidity
1	66	M	Apex	Asystole	Hypertension
2	42	M	Apex	Asystole	Hemochromatosis
3	69	M	RVOT	AV-block	–
4	54	M	RVOT	Chronotropic incompetence	–

Written informed consent was obtained from all participants. This study complied with the declaration of Helsinki and received approval from the local ethical committee and the institutional scientific board.

#### Body surface potential mapping

Three different electrode layouts were used to record data in the healthy volunteers.

*Configuration I* 62 electrodes surrounding the thorax.

*Configuration II* 62 concentrated (30 mm inter-electrode distance) electrodes directly overlaying the heart.

*Configuration III* 62 super-concentrated (20 mm inter-electrode distance) electrodes directly overlaying the heart.

BSP were recorded using a 65-channel (62 thorax electrodes) ActiveTwo BSPM system with passive electrodes and shielded cables (BioSemi BV, Amsterdam, The Netherlands). A sampling rate of 2048 Hz was selected and every data acquisition was performed for 60 s.

In the pacemaker patients, BSP were recorded using the 62 super-concentrated electrode configuration (configuration III). Potentials were recording during right ventricular (RV) pacing at a rate exceeding the intrinsic rate with at least 15 beats/min (paced AV-delay 70 ms).

Every BSP recording was immediately followed by an MRI scan in order to register the electrode positions to the anatomy of the volunteer.

#### Magnetic resonance imaging

After each BSPM recording, MRI markers were applied to replace all torso electrodes. These markers were used to locate the electrode positions on the MRI images, thereby minimizing the systematic error in the inverse procedure.

Axial, coronal and sagittal anatomical images were obtained using a Turbo Spin Echo (black blood) sequence during breath hold (slice thickness 6 mm, no gap between slices).

MRI was performed on a Siemens Aera 1.5 Tesla MRI scanner (Siemens Healthcare, Erlangen, Germany).

For patients with an implanted pacemaker, pacing thresholds, P- and R-wave amplitude and lead impedance were determined before entering the MRI room and the pacemaker system was programmed into MRI SureScan^®^ mode [17]. These parameters were again determined after the examination and compared to the initial values. Finally, original programming of the pacemaker was restored.

#### Inverse reconstruction of recorded ECG data

From the MRI images, a 3D thorax model was constructed comprising the epicardial surface and the thorax volume conductor, accounting for lungs, liver and spleen. Epicardial potentials were calculated from the recorded BSP (*P*_bs_) using *P*_epi_ = (*T*^*T*^*T* + *λ*^*2*^*I*)^−1^*T*^*T*^*P*_bs_, where *T* is the forward transfer matrix and *λ* is the regularisation strength. Following each BSP recording, epicardial activation sequences were inversely reconstructed and visualized.

### Evaluation of the quality of the inverse results

To evaluate the quality of the results, ECGs were reconstructed from the inverse by forward transformation. The correlation between the recorded ECG potentials and the computed ECG potentials was subsequently determined.

Note that while an a priori activation model was used for simulations to optimize the electrode positioning, no such model was used to perform the inverse reconstruction from recorded human BSP.

### Patients with an implanted pacemaker system

#### Localization error

An investigator blinded to the actual ventricular lead tip position, identified the site of earliest depolarization on the colour-coded epicardial potential map. Subsequently, the distance between this site and the position of the ventricular lead tip on the MRI images was determined. Hence, the localization error was quantified as the distance between the true pacing location and the pacing location projected from the inverse.

#### Amount of true detail

The amount of detail was evaluated by performing a threshold-test on the epicardial potential peak induced by a pacing stimulus at a well-known electrode location. When the threshold was set too high, the potential peak would split, suggesting false detail.

**Fig. 4:**
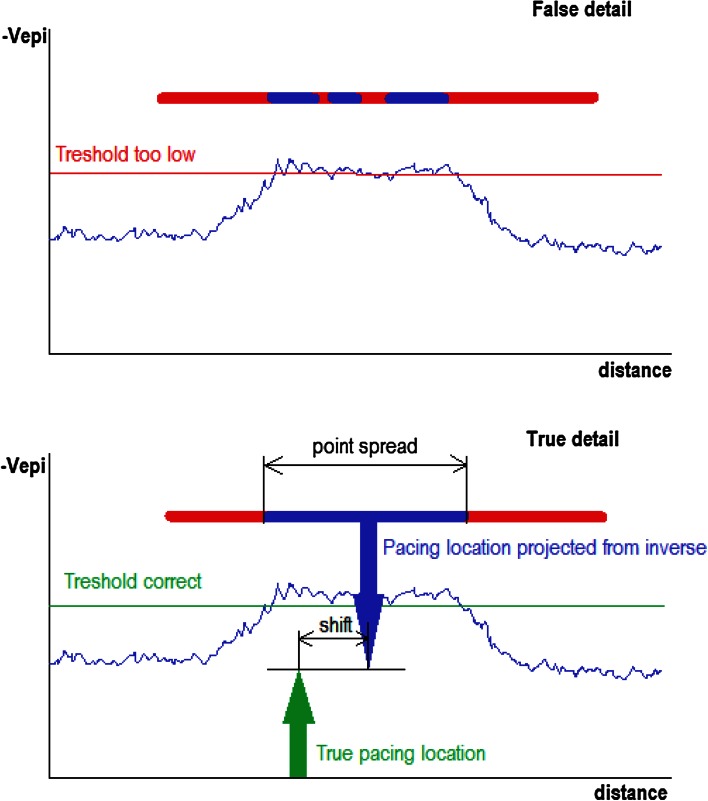
The amount of detail was evaluated by performing a threshold-test on an epicardial potential peak induced by a pacing stimulus. When the threshold is set too high, the potential peak will split, suggesting false detail (*upper panel*). The highest threshold value that does not cause the potential peak to split, reflects the minimum amount of true detail in the inversely reconstructed potential (*lower panel*)

The minimum size of the inversely mapped potential peak induced by pacing is determined by the highest threshold value that does not cause the peak to split. The detail shown in this case is true, rather than false (Fig. 4).

The smallest visible detail was quantified as the maximum point spread cross-section in mm of the potential peak due to pacing.

### Computing platform

All analyses were performed on a 2.4 GHz quadcore laptop running the Windows 8 OS. Solving the potential equations was delegated to an Ubuntu 12.10 virtual machine running on this laptop. Correlation coefficients were determined using Pearson’s product moment correlation coefficients as computed by the NumPy library.

## Results

### Computer simulations

#### 252 electrodes surrounding the thorax

**Fig. 5:**
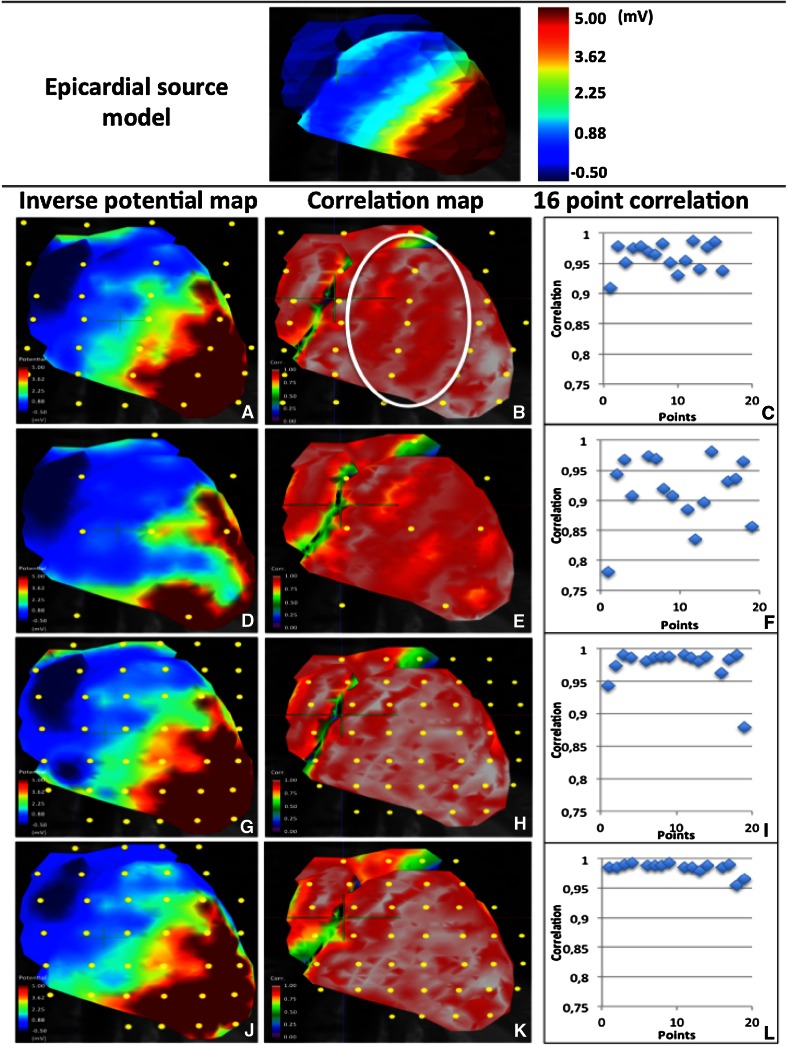
Epicardial source model at 30 ms into the QRS (*upper panel*). Epicardial potential map at 30 ms into the QRS, temporal correlation map and correlation plot of 16 sampling points for every simulated electrode layout (*lower panel*). **a**–**c** 252 electrodes surrounding the thorax, **d**–**f** 62 surrounding the thorax, **g**–**i** 62 concentrated, **j**–**l** super-concentrated. A reduced correlation was observed in areas between two electrodes (*encircled* in **b**). The highest correlation with the epicardial source model was obtained with the super-concentrated electrode placement

This electrode layout provided a high image quality (Fig. 5a, video 1). Right ventricular breakthrough could be easily discerned. An overall high correlation (*R* = 0.96; *p* < 0.01) with the source model was found. The correlation map (Fig. 5b) clearly demonstrated a reduced correlation in areas with increased electrode spacing.

#### 62 electrodes surrounding the thorax

This electrode configuration resulted in a poor image quality (Fig. 5d, video 2). Several gaps appeared in the epicardial potential map, indicating loss of information in these areas. A reduced overall correlation compared to the source model was found (*R* = 0.92; *p* < 0.01) (Fig. 5e).

#### 62 electrodes concentrated (30 mm inter-electrode distance)

When concentrating all available electrodes on the anterior part of the thorax in the region directly overlaying the heart, a clinical relevant image of the potentials on the anterior epicardium was obtained (Fig. 5g, video 3). Figure 5h shows that the correlation with the source model greatly improved (*R* = 0.97; *p* < 0.01), compared to the configuration using 62 electrodes surrounding the thorax.

#### 62 electrodes super-concentrated (20 mm inter-electrode distance)

By reducing the inter-electrode distance to 20 mm, image quality improved. The depolarization front appeared to be more homogeneous (Fig. 5j, video 4). This was confirmed by a slightly higher correlation with the source model (*R* = 0.98; *p* < 0.01), compared to that obtained using the 30 mm electrode spacing configuration. As can be observed in Fig. 5k, l, IPM using the super-concentrated electrode configuration provided the highest correlation with the source model.

### Inverse reconstruction of recorded human data

The BSP recording and MRI examination lasted approximately 60 min. Segmentation and data processing lasted approximately 150 min. Correlation coefficients between measured and reconstructed ECGs were >0.94 for all leads used in the inversion and >0.97 for 85 % of those leads.

#### 62 electrodes surrounding the thorax

The 62 electrodes surrounding the thorax did not provide clinical sufficient information (video 5). Only ventricular epicardial activation could be reconstructed using this configuration. Regions with no or low signal variance were observed as gaps in the reconstructed epicardial potentials.

#### 62 electrodes concentrated (30 mm inter-electrode distance)

Concentrated positioning of the 62 available electrodes, directly above the heart, improved the overall resolution (video 6). Although reduced in size and number, areas of low signal were still present when using this electrode configuration.

#### 62 electrodes super-concentrated (20 mm inter-electrode distance)

**Fig. 6:**
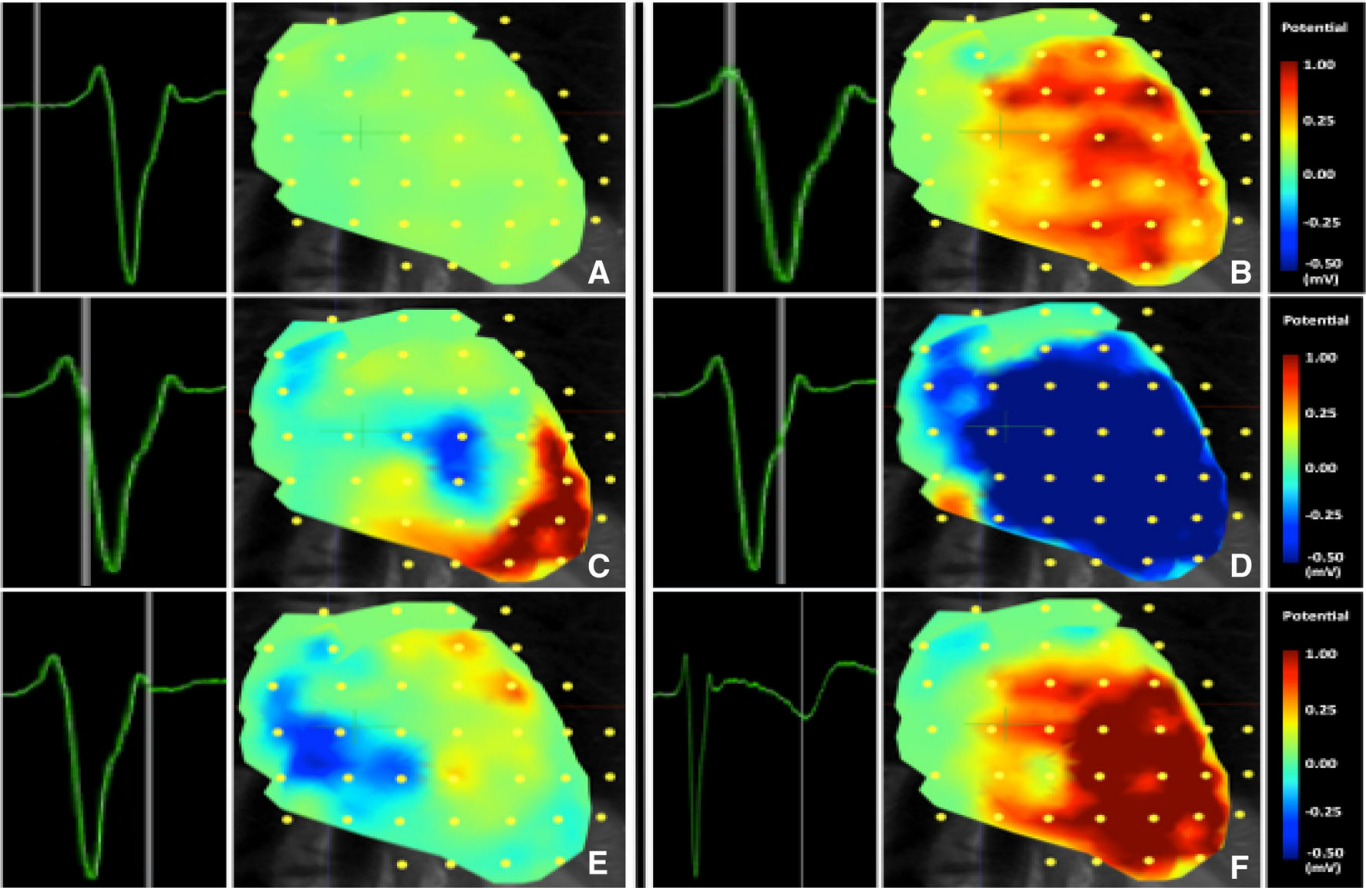
Epicardial potentials during a QRST interval inversely estimated from human BSP recorded using the super-concentrated (20 mm inter-electrode distance) electrode configuration. Right ventricular breakthrough can be observed in panel **c**

Higher concentration of the electrode configuration (20 mm inter-electrode distance) resulted in a substantial increase of image resolution (video 7). Atrial and ventricular activation could be clearly distinguished and spatially localized in the reconstructed epicardial activation sequence. Figure 6 shows epicardial potential maps for six instants of time during the QRST interval. In all three volunteers, similar results were obtained.

### Reconstruction of pacing sites

None of the patients reported any complaints during or after the MRI examination. Pacing thresholds and leads impedances remained unaffected by the MRI scan in all patients.

For all patients, the site of earliest ventricular depolarization could be identified. In two patients, depolarization started in the superior part of the right ventricular septum. In remaining patients, the site of earliest depolarization was located in the apical region of the right ventricle.

### In vivo evaluation of localization error and amount of detail

A mean localization error of 5.3 mm was found. The mean size of the smallest visible detail during pacing, determined by a threshold test, was 7 mm. Individual values are listed in Table 2.

**Table 2:** Localization errors and amount of detail in pacemaker patients

	Localization error (mm)	Max point spread (mm)
Patient 1 (RV apex)	5	7
Patient 2 (RV apex)	5	8
Patient 3 (RVOT)	5	7
Patient 4 (RVOT)	6	6

## Discussion

This study investigated the optimal positioning of only 62 torso electrodes for IPM in the absence of an a priori model. Computer simulations were used to improve insight and predict image quality of different electrode configurations. A configuration of 62 electrodes positioned on the anterior part of the thorax, with a 20 mm inter-electrode distance, provided the highest amount of detail in the epicardial potential maps of the anterior side of the heart.

In addition, the epicardial potential distribution reconstructed using this configuration demonstrated the highest correlation (*R* = 0.98; *p* < 0.01) with the original epicardial source model. Using this configuration, a minimum occurring at 10 ms into the QRS near V1, reflecting right ventricular breakthrough could be discerned. This finding is in accordance with previous observations reported by Taccardi in 1963 [18] and Okamoto et al. in 1990 [19].

The results of application of this method in patients with implanted pacemakers indicated a clinically relevant reconstruction quality. A mean localization error of 5.3 mm was found in the pacemaker patients.

### Clinical relevance of this study

Inverse potential mapping is a promising but also challenging modality to gain further insight into cardiac substrates and arrhythmia mechanisms in a non-invasive fashion. This study focused on simplification of the procedure by applying a reduced number of recording electrodes. The ventricular paced beats analyzed in this study served as ectopic ventricular foci. The mapping approach presented in this paper may help to tailor the invasive electrophysiological procedure to the individual patient. The concentrated electrode configuration may make it an attractive clinical alternative in situations where this specific view is required.

### Importance of simulations

The possibility to simulate epicardial potentials from random electrode configurations facilitated a stepwise approach towards optimal electrode positioning. In this way, the simulations guided the placement of the electrodes. In addition, the validity of the simulations could be determined by application of the selected configurations in vivo. The validity of the simulations was subsequently confirmed by the results obtained in patients with implanted pacemaker systems.

### A priori model

**Fig. 7:**
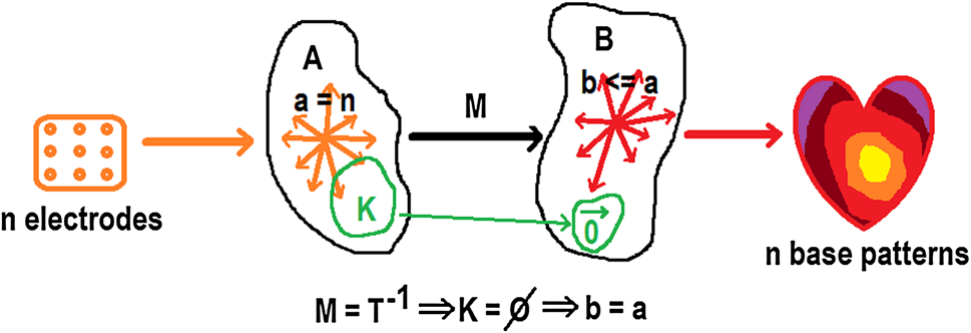
Linear mapping *M* denotes the ideal reconstruction, reflecting the unknown but exact linear quasi-stationary field equations. *M* maps *n* independent electrode signals onto at most *n* independent epicardial base vectors. Note that, by definition of *M*, no reconstruction method can do better. In practice, ill conditioning will render certain subsets of epicardial patterns indistinguishable, reducing the dimension of the solution space

In literature [20–22], inverse procedures involving 64 or fewer electrodes always apply an a priori activation model. Although detailed images may be obtained using even <20 electrodes, the number of degrees of freedom in this situation fundamentally limits the number of pathology related activation patterns that can be represented. This is further elaborated upon in appendix and in Fig. 7.

Obtaining detailed images of cardiac surface potentials using a limited number of electrodes, without excluding a wide variety of pathological activation patterns by constraining the solutions using an a priori activation model derived from healthy myocardial tissue, requires focusing all degrees of freedom on a limited region of interest on the cardiac surface.

### MRI

Although computed tomography (CT) is frequently preferred due to the speed of the acquisition process, MRI allows reliable function analysis, assessment of wall motion abnormalities and highly detailed characterization of tissue [23–26]. Unlike CT, MRI does not use radiation. Hence, MRI is the preferred imaging modality to be repeatedly used in patients. In recent years, the safe performance of MRI in patients with non-MRI-conditional pacing devices has been demonstrated [27–29]. In addition, MRI-conditional devices have been introduced, decreasing the risk of potential hardware or software interactions [30].

### Limitations of this study

Application of 62 anterior electrodes with an inter-electrode distance of 20 mm enables detailed reconstruction of an anterior view of the epicardial potentials in the absence of an a priori model. Information on the posterior area of the heart could not be reconstructed from the BSP recorded using the anterior positioned super-concentrated electrode configuration. By increasing the total number of recording electrodes and by positioning electrodes on the back of the thorax this can be resolved. But since the application of a large number of electrodes is time consuming, implementation in the clinical arena may still be challenging. Hence, an optimal balance between information content and clinical utility is pursued. Parallel computation of the inverse solution will further reduce the post-processing time.

In the presence of a limited number of electrodes, electrode positioning is crucial. In order to achieve a high resolution, it is very important to position the electrodes directly overlaying the heart. Because this may be difficult to determine, a rapid exploratory MRI scan (scout anatomical images) prior to BSPM may help to optimize electrode positioning. Regarding the small number of patients in this study, further research is needed to further evaluate the clinical benefits of this non-invasive mapping strategy.

### Future perspective

Although IPM is considered a promising technique to complement conventional invasive electrophysiological procedures, it has not yet advanced to routine clinical application. This is mainly due to the time consuming nature of the acquisition and post-processing of the data. The possibility to derive detailed information on cardiac excitation from a rapid and simplified BSPM procedure may facilitate clinical implementation. The ability to perform detailed simulations using patient data may provide clinicians valuable insight into the potential impact of their treatment. Non-invasive characterization of arrhythmogenic foci or substrates, prior to invasive electrophysiological or device implant procedures, may help to increase therapeutic outcome. Further research is required to provide evidence of the effectiveness and accuracy of this approach to IPM.

## Conclusion

The purpose of this study was to investigate the feasibility of IPM using only 62 torso electrodes without the aid of an a priori model. By concentrating the available electrodes in the area directly overlaying the heart, a high-resolution anterior view of the epicardial potentials can be obtained. Application of this mapping approach in patients with implanted MRI-conditional pacemakers demonstrated a clinically relevant inverse reconstruction accuracy. Further research needs to be performed to further evaluate the clinical benefits of this technique.

## Electronic supplementary material


**Video 1.** Epicardial potential map of a simulation using 252 electrodes surrounding the thorax. This configuration served as a reference (MP4 2007 kb)

**Video 2.** Epicardial potential map of a simulation using 62 electrodes surrounding the thorax (MP4 2425 kb)

**Video 3.** Epicardial potential map of a simulation using 62 electrodes (30 mm inter-electrode distance) directly overlaying the heart (MP4 1881 kb)

**Video 4.** Epicardial potential map of a simulation using 62 electrodes (20 mm inter-electrode distance) directly overlaying the heart (MP4 2481 kb)

**Video 5.** Inversely reconstructed epicardial potential map of a BSP recording using 62 electrodes surrounding the thorax (MP4 2144 kb)

**Video 6.** Inversely reconstructed epicardial potential map of a BSP recording using 62 electrodes (30 mm inter-electrode distance) directly overlaying the heart (MP4 2453 kb)

**Video 7.** Inversely reconstructed epicardial potential map of a BSP recording using 62 electrodes (20 mm inter-electrode distance) directly overlaying the heart (MP4 1859 kb)

## Appendix

Let *n* be the number of electrodes, let a be the dimension of the linear span *A* of the electrode potential vectors and let *b* be the dimension of the linear span *B* of the reconstructed epicardial potential vectors. Then *a* = *n* and *b* ≤ *a*, since the base vectors of *B* are, by linearity of the quasi-stationary electrical field equations, obtained by a linear mapping *M* from the base vectors of *A*, that may have a kernel *K* of dimension *k* > 0. In general *b* = *a*, since *M* will be non-degenerate as is illustrated in Fig. 7. Note that the exact nature of the parameter estimation procedure plays no role in this fundamental relationship.

## Abbreviations

BSP: Body surface potentials
BSPM: Body surface potential mapping
CT: Computed tomography
ECG: Electrocardiogram
EP: Electrophysiological
IPM: Inverse potential mapping
MRI: Magnetic resonance imaging
